# Psychometric benefits of adding bolt-ons to the EQ-5D-5L in populations undergoing minimally invasive cosmetic procedures

**DOI:** 10.1007/s10198-025-01772-9

**Published:** 2025-03-13

**Authors:** Eszter Mercédesz Müller, Anna Nikl, Máté Krebs, Péter Holló, Valentin Brodszky, Lajos Vince Kemény, Fanni Rencz

**Affiliations:** 1 Department of Dermatology, Venereology and Dermatooncology, Semmelweis University, 42 Mária utca, Budapest, H-1085 Hungary; 2 Károly Rácz Conservative Medicine Division, Semmelweis University Doctoral School, 26 Üllői út, Budapest, H-1085 Hungary; 3 Department of Health Policy, Corvinus University of Budapest, 8 Fővám tér, Budapest, H-1093 Hungary; 4 Department of Physiology, Semmelweis University, 37-47 Tűzoltó utca, Budapest, H-1094 Hungary; 5 HCEMM-SU Translational Dermatology Research Group, Semmelweis University, 37-47 Tűzoltó utca, Budapest, Budapest, H-1094 Hungary

**Keywords:** Minimally invasive cosmetic procedures, EQ-5D, Bolt-ons, Health-related quality of life, Psychometric properties, I10

## Abstract

**Objectives:**

There is growing interest in measuring health outcomes associated with minimally invasive cosmetic procedures (MICPs), such as botulinum toxin and hyaluronic acid injections. However, the EQ-5D may have limited content validity for this purpose. This study aims to psychometrically test five additional dimensions (‘bolt-ons’) for the EQ-5D-5L in individuals planning or undergoing MICPs.

**Methods:**

In 2023, a cross-sectional, online survey was conducted with 149 women planning MICPs and 215 who had recently undergone them. Respondents completed the EQ-5D-5L, five bolt-ons (skin irritation, self-confidence, sleep, social relationships, tiredness), the Rosenberg Self-Esteem Scale (RSES) and the Brief Fear of Negative Evaluation Scale-Straightforward Items (BFNE-S). The following psychometric properties were tested for the EQ-5D-5L + bolt-on(s): ceiling, convergent and divergent validity, explanatory power and known-groups validity.

**Results:**

Adding tiredness (22%), self-confidence (23%) or sleep bolt-ons (27%) substantially reduced the ceiling of the EQ-5D-5L (47%). The self-confidence and social relationships bolt-ons showed a moderate or strong correlation with the RSES and BFNE-S total scores (-0.462 to -0.679). The tiredness and self-confidence bolt-ons improved the EQ-5D-5L’s explained variance in EQ VAS scores from 37% to 45%. The self-confidence and social relationships bolt-ons improved the EQ-5D-5L’s discrimination between known groups based on self-esteem and bodily appearance (relative efficiency: 2.72 to 2.82).

**Conclusions:**

Relevant bolt-ons substantially enhance the psychometric performance of the EQ-5D-5L in MICP populations. The self-confidence and tiredness bolt-ons may be recommended as primary choices for use alongside the EQ-5D-5L, both in clinical studies and as part of sensitivity analyses in economic evaluations of MICPs.

**Supplementary Information:**

The online version contains supplementary material available at 10.1007/s10198-025-01772-9.

## Introduction

In recent years, minimally invasive cosmetic procedures (MICPs) have grown in popularity, and a continuous rise can be observed in the number of procedures performed worldwide [1]. MICPs involve methods such as various injectable treatments, lasers, and chemical peels [2]. Unlike cosmetic surgery, these office-based interventions have less downtime [3]. The most commonly performed MICPs are botulinum toxin and hyaluronic acid injections [1]. According to a recent survey by the International Society of Aesthetic Plastic Surgery conducted in 23 countries, 85% of MICP users are female [1]. However, no country-specific data is available for Hungary. Many individuals are motivated to seek cosmetic procedures for various reasons, such as improving mental health and confidence in social settings and enhancing cosmetic appearance [4, 5]. Several studies demonstrated that MICPs result in an improved health-related quality of life (HRQoL). MICPs, for example, have been reported to improve mental health, self-esteem, body image, appearance-related distress and social functioning in healthy individuals who voluntarily underwent these treatments [6–11].

Accurate measurements of HRQoL using validated instruments in the context of MICPs is important not only for clinicians to monitor patients’ clinical status but also because HRQoL findings can provide valuable information about treatment benefits, which can be used to demonstrate the value of these procedures and inform economic evaluations. This is particularly important as, generally, treatments for cosmetic reasons are not covered by health insurance in most countries unless deemed medically necessary (e.g., reconstruction) [12]. The EQ-5D is the most commonly used generic preference-accompanied HRQoL measure to estimate quality-adjusted life years in cost-utility analyses of health interventions, recommended in numerous national health technology assessment guidelines [13, 14]. It has demonstrated good validity and responsiveness across various patient populations and cultures [15]. However, developed as a generic instrument [16], the EQ-5D may not cover all important dimensions of HRQoL for specific populations [17]. Additional dimensions (‘bolt-ons’) can be added to an instrument to enhance its sensitivity [18]. In a recent systematic review, 26 different EQ-5D bolt-ons were identified, with the most frequently mentioned or tested bolt-ons being cognition, vision, relationships, sleep, hearing, energy and tiredness [19]. In patients with psoriasis, two bolt-ons were developed: skin irritation and self-confidence [20]. Adding skin irritation and self-confidence bolt-ons to the EQ-5D improved its psychometric performance in patients with chronic dermatological diseases, such as psoriasis [20–22], atopic dermatitis [23], urticaria [24] and genodermatoses [25]. These bolt-ons may be relevant beyond these conditions, such as for a population seeking MICPs. Given that improving mental and social aspects of HRQoL is an important self-reported motivation for individuals considering cosmetic procedures [4]; the social-relationships, tiredness and sleep bolt-ons are also deemed relevant considering their potential association with psychological health [26]. This is further supported recent findings, suggesting that vitality (a positively formulated version of tiredness bolt-on), sleep, social-relationships, and community connectedness bolt-ons providing useful additon to the EQ-5D by capturing psychosocial aspects of HRQoL [27, 28]. Furthermore, fatigue has been described as a well-known potential adverse effect of some of these MICPs (e.g. botulinum toxin injections) [29].

To date, no bolt-ons have been psychometrically tested in populations planning or undergoing MICPs. Our aim, therefore, was to investigate the psychometric properties of the five-level EQ-5D (EQ-5D-5L) with potentially relevant bolt-on dimensions in this population.

## Methods

### Study design and patient population

An online cross-sectional questionnaire survey was conducted in Hungary between February and December 2023. Permission to carry out the study was granted by the Semmelweis University Regional and Institutional Committee of Science and Research Ethics under reference no. SE/RKEB: 15/2023. To be eligible for the study, individuals were required to (i) have undergone MICP(s) (‘undergone group’) in the last two months or to plan to undergo such procedure(s) (‘planning group’) in the next two months (e.g., soft tissue augmentation, botulinum toxin treatments, mesotherapy, monofilament thread therapy, cog thread lifting, facial laser resurfacing, medical grade peeling and platelet-rich plasma treatment); (ii) be 18 years or older; (iii) be able to read and understand the questions in Hungarian; and (iv) provide informed consent. Patients were recruited via social media groups and personal contacts of the research team. These social media groups provide a platform for individuals who want to share their experiences about cosmetic surgery and MICPs. We shared the questionnaire in six groups (with member counts ranging from 1000 to 60,000). Moreover, the survey was shared on the social media platform of a large Hungarian women’s lifestyle magazine.

The survey consisted of four parts. The first part included questions about the respondent’s general health status and HRQoL. This section contained a five-point general health scale (excellent-to-poor), the EQ-5D-5L descriptive system, EQ Visual Analogue Scale (EQ VAS), and the skin irritation and self-confidence bolt-ons. The second part collected information regarding the completed or planned aesthetic treatments, including the motivations for seeking MICPs and the procedure’s type, date, and localization. To explore motivations for seeking MICPs, participants were asked to rate their agreement with nine potential reasons for undergoing the procedure on a scale from 1 (completely disagree) to 10 (completely agree), using the same statements as those in a previous study [30]. The only difference between the questionnaires for individuals planning MICPs versus those who had already undergone them was that the latter group received additional questions regarding the adverse effects of the procedures. The third section further asked about the HRQoL and broader wellbeing of patients, including the social relationships, tiredness and sleep bolt-ons for the EQ-5D-5L, Rosenberg Self-Esteem Scale (RSES), Brief Fear of Negative Evaluation Scale – Straightforward Items (BFNE-S), and one question on bodily appearance [question 11 of the World Health Organization Quality of Life Brief Version (WHOQOL–BREF)]. The bolt-ons were presented in separate sections because the EQ-5D-5L with skin irritation and self-confidence bolt-ons, together, functioned as a pilot instrument under development, while the other bolt-ons were exploratory modifications at the time of the survey.

The final section gathered sociodemographic data and medical history, such as age, sex, place of residence, education, chronic diseases, and employment status. The order of the standardized questionnaires and all questions was fixed. All survey questions were compulsory therefore respondents could not proceed to the next question without answering the preceding one. Participation was voluntary and anonymous, and no incentives were offered. The survey was programmed in Qualtrics (Qualtrics 2020, Provo, UT, USA). The questionnaire was tested by members of our research team, based on which we determined that a minimum of six minutes is required for respondents to carefully read and comprehend its content. Speeding respondents (those completing the survey in less than six minutes) were excluded before the data analysis.

### Outcome measures

#### EQ-5D-5L and bolt-ons

The EQ-5D-5L is generic preference-accompanied measure of HRQoL with two parts, a descriptive system consisting of five dimensions and the EQ VAS [16]. The five single-item dimensions include mobility, self-care, usual activities, pain/discomfort, and anxiety/depression. Each dimension has five response levels: 1 = no problems, 2 = slight problems, 3 = moderate problems, 4 = severe problems, and 5 = extreme problems/unable to. The EQ VAS is a vertical scale ranging from 0 (the worst health you can imagine) to 100 (the best health you can imagine). The descriptive system is able to describe a maximum of 5^5^=3125 possible profiles. In this study, five additional, previously developed bolt-on dimensions were used for the descriptive system, including skin irritation, self-confidence, sleep, social relationships and tiredness (Online Resource 1) [20, 31–33]. A 5-level version was used for each bolt-on, similar to the EQ-5D-5L items.

The skin irritation and self-confidence 5-level bolt-ons were developed for patients with psoriasis through literature review, qualitative interviews with patients, and consultation with a clinical expert [20]. The sleep, tiredness and social relationhsips bolt-ons were initially developed as 3-level versions based on the literature and items of other generic preference-accompanied measures and were later extended to 5-level versions [34–37]. All five bolt-ons with the same or slightly different wording have since been psychometrically validated through qualitative and quantitative methods in multiple studies and populations [21–25, 33, 38–40]. While the EQ-5D-5L has a Hungarian value set, index values were not computed in the present study due to the psychometric nature of the research [41].

#### Rosenberg Self-Esteem Scale (RSES)

The validated Hungarian version of the RSES was administered [42]. The RSES is the most widely-used measure of self-esteem that includes ten items, each rated on a four-point Likert scale (strongly agree, agree, disagree, and strongly disagree), whereby items 2, 5, 6, 8, and 9 are reverse-scored [43, 44]. The total score ranges between 0 and 30, where higher scores represent higher self-esteem. A score below 15 indicates low self-esteem [45].

#### Brief Fear of Negative Evaluation Scale – Straightforward Items (BFNE-S)

We used the validated Hungarian version of the BFNE-S questionnaire [46]. BFNE-S is an eight-item questionnaire that evaluates the degree to which an individual feels anxious about being judged negatively. Respondents are asked to rate each item using a 5-point Likert scale (from “not at all characteristic of me” to “entirely characteristic of me”). The total scores range from 0 to 32, with higher scores indicating worse fear of negative evaluation [47].

#### Bodily appearance question

We included one question from the WHOQOL-BREF general quality of life measure for the sake of known group validity tests (“Are you able to accept your bodily appearance?”) [48]. The item is rated on a 5-point Likert-scale (Not at all, A little, Moderately, Mostly, Completely).

### Statistical analyses

We followed an analytical framework described in previous bolt-on studies [18, 33], which included analyses both at level of dimensions and instrument (i.e. EQ-5D-5L with and without bolt-ons). All statistical analyses were conducted using R Statistical Software (version 4.3.2; R Foundation for Statistical Computing, Vienna, Austria). Significance level was set at 0.05 for all analyses.

#### Distributional characteristics, ceiling and informativity

Descriptive statistics were used to summarise the sociodemographic and clinical characteristics of the patients. The characteristics of the ‘planning group’ and the ‘undergoing group’ were compared using Pearson’s Chi-square test (with Yates correction where applicable) for categorical variables, and Student’s t-test for continuous variables. The relative frequencies of patients’ responses for different levels of EQ-5D-5L, bolt-ons, RSES, and BFNE-S were computed. To assess the ceiling, the proportion of patients having ‘no problems’ on one or all dimensions was computed. Both absolute (percentage point, pp) and relative (%) reductions in ceiling were determined after adding the bolt-ons. We also computed the frequency of different health state profiles for the EQ-5D-5L (+ bolt-ons). Informativity was evaluated using Shannon’s absolute informativity (H’) and Shannon’s evenness (relative informativity, J’) indices. We first assessed the informativity for each bolt-on item (item-level informativity). Then, we assessed the informativity of the EQ-5D-5L with and without the bolt-on dimensions (instrument-level informativity) [49, 50]. H’ can be defined as

$$\:{H}^{{\prime\:}}=\:-\sum\:_{i=1}^{L}{p}_{i}*{\text{log}}_{2}{p}_{i}$$,

where *L* is the number of levels in a dimension, and $\:{p}_{i}$ is the percentage of patients in the *i*th level (*i* = 1,…, *L*). J’ measures the evenness of distribution and can be calculated as follows:

$$\:{J}^{{\prime\:}}=\:\frac{H{\prime\:}}{{H{\prime\:}}_{max}}\:,\:\text{w}\text{h}\text{e}\text{r}\text{e}\:{H{\prime\:}}_{max}={\text{log}}_{2}L.$$

Higher H’ and J’ values indicate better informativity, where H’ ranges from 0 to $\:{\text{log}}_{2}L$ and J’ ranges from 0 to 1. We hypothesized that adding any bolt-ons would result in a reduction in ceiling and an improvement in informativity of the EQ-5D-5L.

#### Convergent and divergent validity

Spearman’s rank-order correlations (r_s_) were used for convergent and divergent validity analyses. The divergent validity of all five bolt-ons was examined with the core five EQ-5D-5L dimensions. We hypothesized (very) weak correlations between the bolt-ons and the core EQ-5D-5L dimensions with the exception of the self-confidence and social relationships bolt-ons and the EQ-5D-5L’s anxiety/depression dimension, where we anticipated moderate or strong correlation [21, 23, 33]. Convergent validity was investigated for the self-confidence and social relationships bolt-ons and the EQ-5D-5L’s anxiety/depression dimension with the items and total scores of RSES and BFNE-S, which intend to measure a similar construct. We hypothesized at least moderate correlations between the bolt-ons and the item and total scores of RSES and BFNE-S, with stronger correlations expected for the self-confidence bolt-on [51]. Correlations of the bolt-ons with EQ VAS were also investigated, with the strongest correlations being expected with the tiredness bolt-on [33, 38]. The absolute value of the correlation coefficient (|r_s_|) was interpreted as follows: very weak (< 0.20), weak (0.20–0.39), moderate (0.40–0.59), strong (0.60–0.79), and very strong (≥ 0.80) correlation [52].

#### Explanatory power

Univariable and multivariable linear regression analyses were used to compare the explanatory power of the EQ-5D-5L and bolt-on dimensions, using EQ VAS as dependent variable. In the univariable models, the association between EQ VAS and each dimension was examined individually. In the multivariable models, the bolt-on performing the best in univariable models was added to the model first, in addition to the five core dimensions. Additional bolt-ons were added as long as they increased the adjusted R².

#### Known-groups validity

Mean level sum scores (LSSs) were computed by adding up the responses on each dimension and standardized into a 0-100 scale to compare the known-group validity of the EQ-5D-5L with bolt-on(s) to the EQ-5D-5L alone. The following seven variables were used to define known groups: bodily appearance question of WHOQOL-BREF (whole sample), self-esteem groups as measured by the RSES (whole sample), respondents who had undergone vs. those who planned to undergo MICPs (whole sample), type of procedure performed in the last two months (‘undergone group’), date of the last procedure (‘undergone group’), body mass index (whole sample), and general health status on 5-point excellent-to-poor scale (whole sample). We hypothesized that respondents who are more accepting of their appearance, have higher self-esteem, have already undergone procedures compared to those planning them, had procedures one to two months ago versus one to two weeks ago, and had a normal body mass index would report better overall HRQoL. We did not have a specific a priori hypothesis regarding which type of procedure (e.g. soft tissue augmentation, thread lifting, collagen induction therapies, or botulinum toxin treatment) would be associated with better or worse HRQoL outcomes. However, we generally hypothesized that differences in HRQoL might exist across these groups. Relative efficiency (RE) was calculated as the ratio of the F-statistic from the analysis of variance, where the EQ-5D-5L was taken as a reference (i.e. denominator) [53]. An F-ratio > 1 indicated that EQ-5D-5 L with bolt-on(s) was more efficient at discriminating between subgroups than the EQ-5D-5L alone. To test whether the F-ratio statistically differs from 1, 95% confidence intervals were estimated using 2000 bootstrap samples with accelerated bias correction. Bolt-ons were added to the EQ-5D-5L as long as their inclusion resulted in a statistically significant improvement in RE.

## Results

### Characteristics of the study population

Out of 942 individuals who opened the questionnaire, 387 completed it (41.0%) (Online Resource 2). We excluded five speeders (i.e., those who completed the survey in less than 6 minutes). Furthermore, the research team has decided to exclude a small number of male (n = 16) and nonbinary (n = 2) respondents to ensure the homogeneity of the sample. Overall, 364 female participants were included in the data analysis. Among them, 215 participants had undergone and 149 participants planned MICPs. The mean age of the total sample was 42.2 (SD = 11.5) years, ranging from 19 to 71 years (Table 1). The mean age of the ‘undergone group’ and ‘planning group’ was similar (42.1 vs. 42.5). The most common chronic diseases among the total sample were allergies (19.5%), thyroid disease (17.6%), and hypertension (9.9%). The sample was highly educated.

**Table 1:** Characteristics of the study population

Variables	Total sample (*n* = 364)		Group 1 (undergone) (*n* = 215)		Group 2 (planning) (*n* = 149)		*p*-value^†^
	Mean or *n*	SD or %	Mean or *n*	SD or %	Mean or *n*	SD or %	
**Age (years)**							
19–24	11	3.0	6	2.8	5	3.4	0.546
25–34	107	29.4	61	28.4	46	30.9	
35–44	84	23.1	55	25.6	29	19.5	
45–54	105	28.8	60	27.9	45	30.2	
55–64	45	12.4	29	13.5	16	10.7	
65+	12	3.3	4	1.9	8	5.4	
**Place of residence**							
Capital	181	49.7	112	52.1	69	46.3	0.075
County town	83	22.8	39	18.1	44	29.5	
Other town	70	19.2	46	21.4	24	16.1	
Village	30	8.2	18	8.8	12	8.1	
**Highest level of education**							
Primary or secondary school	88	24.2	54	25.1	34	22.8	0.615
College/university	276	75.8	161	74.9	115	77.2	
**Employment**							
Full-time/self employed	291	79.9	177	82.3	114	76.5	0.219
Other	73	20.1	38	17.7	35	23.5	
**Body mass index (BMI) (kg/m2)**							
Underweight (below 18.5)	24	6.6	17	7.9	7	4.7	0.343
Normal (between 18.5 and 24.9)	226	62.1	139	64.7	87	58.4	
Overweight (between 25 and 29.9)	62	17.0	36	16.7	26	17.4	
Obese (30 or over)	20	5.5	9	4.2	11	7.4	
Type of procedure(s) performed in the last two months or planned in the next two months *							
Lip augmentation	96	26.4	47	21.9	49	33.2	0.019
Soft tissue augmentation of other part of the face	81	22.3	45	20.9	36	24.5	0.466
Mesotherapy	56	15.4	28	13.0	28	19.0	0.134
Botulinum toxin treatment	161	44.2	99	46.0	62	42.0	0.402
Monofilament thread therapy	20	5.5	4	1.9	16	11.1	*p* < 0.001
COG thread lifting	34	9.3	8	3.7	26	17.8	*p* < 0.001
Facial laser resurfacing	55	15.1	17	7.9	38	25.8	*p* < 0.001
Medical grade peeling	22	6.0	6	2.8	16	10.9	*p* < 0.001
Platelet rich plasma treatment	7	1.9	0	0.0	7	4.9	0.001
Other	21	5.8	14	6.5	7	4.8	0.466
Localisation of the procedure performed in the last two months or planned in the next two months *							
Upper third of the face	227	62.4	143	66.5	84	56.4	0.050
Middle third of the face	152	41.8	92	42.8	60	40.3	0.631
Lower third of the face	190	52.2	98	45.6	92	61.7	0.002
Other	16	4.4	12	5.6	4	2.7	0.185
Adverse reaction(s) observed after the latest procedure *							
No adverse reaction	-	-	145	67.4	-	-	-
Heamatoma	-	-	41	19.1	-	-	-
Pain	-	-	28	13.0	-	-	-
Oedema	-	-	32	14.9	-	-	-
Other	-	-	33	15.3	-	-	-
**Number of procedures in the last two months**							
1	-	-	159	74.0	-	-	-
2	-	-	46	21.4	-	-	-
More than 2	-	-	10	4.7	-	-	-
**Date of the last procedure**							
Within one week	-	-	28	13.0	-	-	-
Within two weeks	-	-	31	14.4	-	-	-
Within one month	-	-	39	18.1	-	-	-
Within two months	-	-	117	54.4	-	-	-
**Total number of procedures performed during the lifetime**							
1–2	-	-	63	29.3	-	-	-
3–5	-	-	72	33.5	-	-	-
6–10	-	-	43	20.0	-	-	-
10–15	-	-	21	9.8	-	-	-
16–20	-	-	3	1.4	-	-	-
More than 20	-	-	13	6.0	-	-	-
**Number of comorbidities**							
0	143	39.3	90	41.9	53	35.6	0.682
1	132	36.3	75	34.9	57	38.3	
2–3	76	20.9	43	20.0	33	22.1	
4	13	3.6	7	3.3	6	4.0	
Most common chronic conditions*							
Allergies	71	19.5	43	20.0	28	17.8	0.775
Thyroid disease	64	17.6	34	15.8	30	19.1	0.287
Hypertension	36	9.9	17	7.9	19	12.1	0.128
Anxiety	27	7.4	18	8.4	9	5.7	0.404
Atopic dermatitis	18	4.9	8	3.7	10	6.4	0.196
Rheumatic disease	17	4.7	12	5.6	5	3.2	0.322
Diabetes	16	4.4	8	3.7	8	5.1	0.451
Asthma or chronic obstructive pulmonary disease (COPD)	16	4.4	11	5.1	5	3.2	0.420
Other dermatologic disease	15	4.1	9	4.2	6	3.8	0.940
Depression	12	3.3	6	2.8	6	3.8	0.726
Cardiovascular disease	10	2.7	5	2.3	5	3.2	0.791
Other disease	44	12.1	23	10.7	21	13.4	0.328
**Rosenberg Self-Esteem Scale total score**	21.0	6.3	21.9	5.7	19.8	6.9	0.002
Rosenberg Self-Esteem Scale total score ≥ 15	302	83.0	192	89.3	110	73.8	< 0.001
Rosenberg Self-Esteem Scale total score < 15	62	17.0	23	10.7	39	26.2	
**Brief Fear of Negative Evaluation Scale-Straightforward Items total score**	29.6	8.6	31.0	7.7	27.6	9.3	< 0.001
**EQ visual analogue scale (EQ VAS) score**	85.3	12.4	86.5	11.1	83.5	13.9	0.028

*RSES* Rosenberg Self-Esteem Scale, *BRFE-S* Brief Fear of Negative Evaluation Scale- Straightforward Items, *EQ VAS* EuroQol visual analogue scale, *SD* standard deviation

* = Respondents could choose more than one response option

The number of respondents who responded that they did not know and/or refused to answer was *n* = 32 (8.8%) for the body mass index (BMI) and *n* = 8 (2.2%) for the diagnosis of any chronic disease

The “other” category of employment status includes part-time employed, unemployed, retired, homemaker and student

Values may range from 0 to 30 on the Rosenberg Self-Esteem Scale, 8 to 40 on the Brief Fear of Negative Evaluation Scale-Straightforward Items, and 0 to 100 on the EQ VAS. Higher scores indicate better status on the Rosenberg Self-Esteem Scale and EQ VAS, while worse status on the Brief Fear of Negative Evaluation Scale-Straightforward Items

†: For categorical variables, Pearson’s chi-squared (with Yates continuity correction, where applicable) was used. For continious variables, Student’s t-test was used

Both in the ‘undergone’ and ‘planning’ groups, the three most important motivations for seeking MICPs were to feel better about oneself (85.6% and 81.2% at least slightly agreed), to look more attractive (74.0% and 78.5% at least slightly agreed), and to look younger (62.8% and 59.7% at least slightly agreed). In the ‘undergone group,’ the most common MICPs were botulinum toxin treatment (46.0%), lip augmentation (21.9%), and soft tissue augmentation of other areas of the face (20.9%). The majority of patients in the ‘planning group’ were planning botulinum toxin treatment (42.0%), lip augmentation (33.2%), and laser resurfacing (25.8%). No significant difference was found between the two groups regarding their sociodemographic data. The mean EQ VAS score was significantly higher in the ‘undergone group’ compared to the ‘planning’ group’ (86.5 vs. 83.5; *p* = 0.028). Furthermore, compared to the ‘undergone group’, substantially more participants in the ‘planning group’ scored less than 15 on the RSES, suggesting low self-esteem (26.2% vs. 10.7%; *p* < 0.001). Overall, 12.6% of the participants reported that they can accept their bodily appearance completely. The item-level responses on the RSES and BFNE-S are shown in Online Resources 3 and 4.

### Distributional characteristics, ceiling, informativity

Table 2 displays the distribution of responses on the EQ-5D-5L and bolt-on dimensions. There were only smaller differences in the distribution of responses between the ‘planning group’ and the ‘underwent group’ on the EQ-5D-5L and bolt-on dimensions. Notably, the ‘planning group’ reported more problems with anxiety/depression (53.0% vs. 35.3%), tiredness (82.6% vs. 68.4%) and sleep (62.4% vs. 49.8%). In the total sample, among the five core dimensions, self-care demonstrated the highest ceiling (99.5%), followed by mobility (91.2%), usual activities (89.6%), pain/discomfort (72.0%), and anxiety/depression (57.4%) (Table 3). Three bolt-ons showed lower ceiling than any of the five core dimensions: tiredness (25.8%), self-confidence (30.8%) and sleep (45.1%). Whereas the ceiling of social relationships (64.6%) and skin irritation (81.9%) were in the range of the EQ-5D-5L dimensions.

**Table 2:** Responses on five EQ-5D-5L dimensions and five bolt-ons

	No problems		Slight problems		Moderate problems		Severe problems		Extreme problems/unable to	
	*n*	%	*n*	%	*n*	%	*n*	%	*n*	%
**Total sample (*n*** = **364)**										
Mobility (MO)	332	91.2	27	7.4	4	1.1	1	0.3	0	0.0
Self-care (SC)	362	99.5	1	0.3	1	0.3	0	0.0	0	0.0
Usual activities (UA)	326	89.6	29	8.0	7	1.9	2	0.5	0	0.0
Pain/discomfort (PD)	262	72.0	90	24.7	9	2.5	1	0.3	2	0.5
Anxiety/depression (AD)	209	57.4	115	31.6	30	8.2	7	1.9	3	0.8
Skin irritation bolt-on (SI)	298	81.9	50	13.7	13	3.6	0	0.0	3	0.8
Self confidence bolt-on (SE)	112	30.8	144	39.6	77	21.2	27	7.4	4	1.1
Social relationships bolt-on (SR)	235	64.6	90	24.7	32	8.8	7	1.9	0	0.0
Tiredness bolt-on (TI)	94	25.8	160	44.0	75	20.6	26	7.1	9	2.5
Sleep bolt-on (SL)	164	45.1	139	38.2	45	12.4	13	3.6	3	0.8
**Group 1 (undergone,** ***n*** = 215)										
Mobility (MO)	196	91.2	16	7.4	2	0.9	1	0.5	0	0.0
Self-care (SC)	214	99.5	0	0.0	1	0.5	0	0.0	0	0.0
Usual activities (UA)	200	93.0	12	5.6	2	0.9	1	0.5	0	0.0
Pain/discomfort (PD)	166	77.2	43	20.0	5	2.3	0	0.0	1	0.5
Anxiety/depression (AD)	139	64.7	53	24.7	20	9.3	3	1.4	0	0.0
Skin irritation bolt-on (SI)	182	84.7	23	10.7	10	4.7	0	0.0	0	0.0
Self-confidence bolt-on (SE)	72	33.5	89	41.4	43	20.0	10	4.7	1	0.5
Social relationships bolt-on (SR)	147	68.4	54	25.1	13	6.0	1	0.5	0	0.0
Tiredness bolt-on (TI)	68	31.6	97	45.1	40	18.6	8	3.7	2	0.9
Sleep bolt-on (SL)	108	50.2	81	37.7	20	9.3	6	2.8	0	0.0
**Group 2 (planning,** ***n*** = **149)**										
Mobility (MO)	136	91.3	11	7.4	2	1.3	0	0.0	0	0.0
Self-care (SC)	148	99.3	1	0.7	0	0.0	0	0.0	0	0.0
Usual activities (UA)	126	84.6	17	11.4	5	3.4	1	0.7	0	0.0
Pain/discomfort (PD)	96	64.4	47	31.5	4	2.7	1	0.7	1	0.7
Anxiety/depression (AD)	70	47.0	62	41.6	10	6.7	4	2.7	3	2.0
Skin irritation bolt-on (SI)	116	77.9	27	18.1	3	2.0	0	0.0	3	2.0
Self-confidence bolt-on (SE)	40	26.8	55	36.9	34	22.8	17	11.4	3	2.0
Social relationships bolt-on (SR)	88	59.1	36	24.2	19	12.8	6	4.0	0	0.0
Tiredness bolt-on (TI)	26	17.4	63	42.3	35	23.5	18	12.1	7	4.7
Sleep bolt-on (SL)	56	37.6	58	38.9	25	16.8	7	4.7	3	2.0

*MO* mobility, *SC* self-care, *UA* usual activities, *PD* pain/discomfort, *AD* anxiety/depression, *SI* skin irritation bolt-on, *SE* self-confidence bolt-on, *SR* social relationships bolt-on, *TI* Tiredness bolt-on, *SL* sleep bolt-on

**Table 3:** Ceiling and informativity of EQ-5D-5L and bolt-ons

	Ceiling		Ceiling [EQ-5D-5L + bolt-on(s)]		Absolute ceiling reduction (pp)	Relative ceiling reduction (%)	Item-level informativity		Informativity [EQ-5D-5L + bolt-on(s)]		Total number of health state profiles		
	*n*	%	*n*	%			Shannon’s index H’	Shannon’s evenness index J’	Shannon’s index H’	Shannon’s evenness index J’	*n*	%	max
**EQ-5D-5L**													
Mobility (MO)	332	91.2	172	47.3	-	-	0.49	0.21	2.99	0.26	44	1.4	3125
Self-care (SC)	362	99.5			-	-	0.05	0.02					
Usual activities (UA)	326	89.6			-	-	0.58	0.25					
Pain/discomfort (PD)	262	72.0			-	-	1.04	0.45					
Anxiety/depression (AD)	209	57.4			-	-	1.45	0.62					
**(EQ-5D-5L +) bolt-ons**													
Skin irritation bolt-on (SI)	298	81.9	148	40.7	6.6	14.0	0.86	0.37	3.66	0.26	63	0.4	15,625
Self-confidence bolt-on (SE)	112	30.8	83	22.8	24.5	51.7	1.88	0.81	4.39	0.32	75	0.5	15,625
Social relationships bolt-on (SR)	235	64.6	139	38.2	9.1	19.2	1.32	0.57	3.93	0.28	66	0.4	15,625
Tiredness bolt-on (TI)	94	25.8	81	22.3	25.0	52.9	1.90	0.82	4.42	0.32	77	0.5	15,625
Sleep bolt-on (SL)	164	45.1	99	27.2	20.1	42.4	1.65	0.71	4.32	0.31	76	0.5	15,625
**EQ-5D-5L + TI + SE**	-	-	39	10.7	36.6	77.3	-	-	5.58	0.34	114	0.1	78,125
**EQ-5D-5L + TI + SE + SL**	-	-	27	7.4	39.9	84.3	-	-	6.48	0.35	163	< 0.1	390,625
**EQ-5D-5L + TI + SE + SL + SR**	-	-	26	7.1	40.2	84.9	-	-	6.07	0.29	198	< 0.1	1,953,125
**EQ-5D-5L + all 5 bolt-ons**	-	-	26	7.1	40.2	84.9	-	-	7.10	0.31	216	< 0.1	9,765,625

*MO* mobility, *SC* self-care, *UA* usual activities, *PD* pain/discomfort, *AD* anxiety/depression, *SI* skin irritation bolt-on, *SE* self-confidence bolt-on, *SR* social relationships bolt-on, *TI* Tiredness bolt-on, *SL* sleep bolt-on, *H’* absolute informativity, *J’*relative informativity, *pp* percentage point, *max* maximum

The ceiling in the EQ-5D-5L (considering all five dimensions combined) was 47.3%. Adding the tiredness, self-confidence, sleep, social relationships, and skin irritation individual bolt-ons to the EQ-5D-5 L reduced the ceiling to 22.3%, 22.8%, 27.2%, 38.2% and 40.7%, respectively. Notably, self-confidence and tiredness together reduced the ceiling to 10.7%. When adding all five bolt-ons to the EQ-5D-5L, the ceiling was reduced to 7.1%. The number of observed health state profiles increased by adding bolt-ons to the EQ-5D-5L, with the largest rise being observed with tiredness (from 44 to 77).

Both absolute (H’) and relative (J’) informativity were higher for the self-confidence, tiredness and sleep bolt-ons than for any of the core EQ-5D-5-L dimensions (Table 3). Absolute informativity increased with the addition of any of the five bolt-ons to the EQ-5D-5L. This was also true for relative informativity, except for skin irritation.

### Convergent and divergent validity

In line with our hypotheses, moderate correlation was observed between self-confidence and anxiety/depression (r_s_=0.515), and between social relationships and anxiety/depression (r_s_=0.439) (Table 4). Tiredness, skin irritation and sleep bolt-ons exhibited only weak or very weak correlations with any of the five core dimensions. Tiredness was moderately correlated with EQ VAS (r_s_ =-0.415), while self-confidence, social relationships and sleep exhibited weak correlation (range of r_s_: -0.293 to -0.321).

**Table 4:** Correlations between the EQ-5D-5L and bolt-on dimensions and EQ VAS

Dimension/scale	Mobility (MO)	Self-care (SC)	Usual activities (UA)	Pain/discomfort (PD)	Anxiety/depression (AD)	EQ VAS	Skin irritation bolt-on (SI)	Self-confidence bolt-on (SE)	Social relationships bolt-on (SR)	Tiredness bolt-on (TI)	Sleep bolt-on (SL)
Mobility (MO)	-	-	-	-	-	-	**-**	-	-	-	-
Self-care (SC)	0.120	-	-	-	-	-	-	-	-	-	-
Usual activities (UA)	0.342	0.227	-	-	-	-	-	-	-	-	-
Pain/discomfort (PD)	0.372	0.138	0.473	-	-	-	-	-	-	-	-
Anxiety/depression (AD)	0.093*	0.126	0.309	0.357	-	-	-	-	-	-	-
EQ VAS	-0.282	-0.114	-0.329	-0.426	-0.373	-	-	-	-	-	-
Skin irritation bolt-on (SI)	0.027*	0.074*	0.127	0.099*	0.111	-0.099*	-	-	-	-	-
Self-confidence bolt-on (SE)	-0.015*	0.043*	0.202	0.242	0.515	-0.321	0.232	-	-	-	-
Social relationships bolt-on (SR)	0.075*	0.107	0.266	0.221	0.439	-0.293	0.112	0.462	-	-	-
Tiredness bolt-on (TI)	0.207	0.101*	0.343	0.373	0.399	-0.415	0.127	0.386	0.355	-	-
Sleep bolt-on (SL)	0.160	0.121	0.211	0.252	0.312	-0.247	0.098*	0.241	0.207	0.393	-

*EQ VAS EQ* Visual Analogue Scale, *MO* mobility, *SC* self-care, *UA* usual activities, *PD* pain/discomfort, *AD* anxiety/depression, *SI* skin irritation bolt-on, *SE* self-confidence bolt-on, *SR* social relationships bolt-on, *TI* Tiredness bolt-on, *SL* sleep bolt-on

* = Spearman’s correlation coefficient p-value ≥ 0.05

The self-confidence bolt-on demonstrated strong correlation with both RSES and BFNE-S total scores (r_s_=-0.679 and − 0.656) (Table 5). The anxiety/depression dimension and social relationships bolt-ons correlated moderately with the same two external measures (range of r_s_ -0.462 to -0.560). The strengths of the correlations of the anxiety/depression dimension and self-confidence and social relationships bolt-ons with the items of RSES and BFNE-S were strong or moderate in 12, 17, and 10 of the 18 correlations, respectively, with the strongest correlations observed for the self-confidence bolt-on across all RSES and BFNE-S items.

**Table 5:** Convergent validity of the anxiety/depression dimension, self-confidence and social relationships bolt-ons

	Anxiety/depression (AD)	Self-confidence (SE) bolt-on	Social relationships (SR) bolt-on
**Rosenberg Self-Esteem Scale**			
On the whole, I am satisfied with myself.	-0.443	-0.589	-0.456
At times I think I am no good at all.	-0.526	-0.566	-0.425
I feel that I have a number of good qualities.	-0.330	-0.406	-0.323
I am able to do things as well as most other people.	-0.343	-0.381	-0.281
I feel I do not have much to be proud of.	-0.349	-0.418	-0.331
I certainly feel useless at times.	-0.504	-0.563	-0.412
I feel that I’m a person of worth, at least on an equal plane with others.	-0.330	-0.493	-0.366
I wish I could have more respect for myself.	-0.412	-0.517	-0.338
All in all, I am inclined to feel that I am a failure.	-0.495	-0.544	-0.445
I take a positive attitude toward myself.	-0.427	-0.581	-0.408
**Total score**	**-0.560**	**-0.679**	**-0.506**
**Brief Fear of Negative Evaluation Scale – Straightforward Items**			
I worry about what other people will think of me even when I know it doesn’t make any difference.	-0.420	-0.556	-0.397
I am frequently afraid of other people noticing my shortcomings.	-0.412	-0.555	-0.419
I am afraid others will not approve of me.	-0.339	-0.450	-0.413
I am afraid that other people will find fault with me.	-0.378	-0.530	-0.410
When I am talking to someone, I worry about what they may be thinking about me.	-0.417	-0.559	-0.438
I am usually worried about what kind of impression I make.	-0.428	-0.584	-0.405
Sometimes I think I am too concerned with what other people think of me.	-0.425	-0.576	-0.390
I often worry that I will say or do the wrong things.	-0.541	-0.608	-0.366
**Total score**	**-0.494**	**-0.656**	**-0.462**

The numbers are Spearman’s correlation coefficients. P-value < 0.05 for all correlation coefficients

### Explanatory power

The results of the univariable linear regression analysis revealed that usual activities (adjusted R^2^ = 0.196) and pain/discomfort (adjusted R^2^ = 0.241) demonstrated the highest explanatory power for the variance of EQ VAS scores among the core EQ-5D-5L dimensions (Table 6). Tiredness (adjusted R^2^ = 0.240) and self-confidence (adjusted R^2^ = 0.181) performed the best among the bolt-ons. Adding two bolt-ons (tiredness and self-confidence) to the EQ-5D-5L improved the explained variance of EQ VAS scores, raising the adjusted R^2^ from 0.369 to 0.445 (Table 6). No further improvement in adjusted R^2^ was achieved after the second bolt-on.

**Table 6:** The explanatory power of EQ-5D-5 L and bolt-ons on EQ VAS

Selection of dimensions	EQ VAS	
	Adjusted *R*^2^	ΣΔ adjusted *R*^2^
**EQ-5D-5L dimensions**		
Mobility (MO)	0.115	-
Self-care (SC)	0.021	-
Usual activities (UA)	0.196	-
Pain/discomfort (PD)	0.241	-
Anxiety/depression (AD)	0.180	-
**Bolt-on dimensions**		
Skin irritation bolt-on (SI)	0.021	-
Self confidence bolt-on (SE)	0.181	-
Social relationships bolt-on (SR)	0.122	-
Tiredness bolt-on (TI)	0.240	-
Sleep bolt-on (SL)	0.086	-
**EQ-5D-5L(+ bolt-on) dimensions**		
MO + SC + UA + PD + AD	0.369	-
MO + SC + UA + PD + AD + TI	0.412	0.043
MO + SC + UA + PD + AD + TI + SE	0.445	0.033

*EQ VAS EQ* Visual Analogue Scale, *MO* mobility, *SC* self-care, *UA* usual activities, *PD* pain/discomfort, A*D* anxiety/depression, *SI* skin irritation bolt-on, *SE* self-confidence bolt-on, *SR* social relationships bolt-on, *TI* tiredness bolt-on, *SL* sleep bolt-on, *R*^*2*^ coefficient of determination

### Known-groups validity

The addition of tiredness bolt-on significantly improved the EQ-5D-5L’s ability to discriminate between groups of participants based on whether they had undergone a MICP or were planning to, with a RE of 2.03 (95%CI 1.25–9.11) (Table 7). The addition of the self-confidence and social relationships bolt-ons significantly increased the known-groups validity concerning the self-esteem groups based on RSES and bodily appearance, with RE values of 2.82 (95%CI 1.96–4.81) and 2.72 (95%CI 1.96–4.71), respectively. For the following known-groups, none of the bolt-ons improved the discriminatory power compared to the EQ-5D-5L: type of procedure performed in the last two months, date of the last procedure, body mass index, and general health status.

**Table 7:** Known-groups validity of the EQ-5D-5L and bolt-ons

	Mean (SD) EQ-5D-5L + bolt-on(s) LSS (0-100)					RE (95% CI*), ref: previous row	RE (95% CI*), ref: no bolt-ons
**Subgroups**	**Group 1 (undergone)**	**Group 2 (planning)**	-				
**n**	**215**	**149**				-	-
EQ-5D-5L	4.72 (7.76)	7.18 (8.67)				-	-
EQ-5D-5L + TI	7.98 (8.61)	12.00 (10.30)				2.03 (1.25–9.11)	2.03 (1.25–9.11)
**Total score on the Rosenberg Self-Esteem Scale (RSES)**	**RSES < 15**	**RSES ≥ 15**					
**n**	**62**	**302**				-	-
EQ-5D-5L	12.3 (8.95)	4.37 (7.38)				-	-
EQ-5D-5L + SE	19.60 (9.35)	7.19 (7.65)				2.23 (1.68–3.44)	2.23 (1.68–3.44)
EQ-5D-5L + SE + SR	21.20 (9.44)	7.32 (7.61)				1.26 (1.08–1.49)	2.82 (1.96–4.81)
**Are you able to accept your bodily appearance?**	**Not at all**	**A little**	**Moderately**	**Mostly**	**Completely**		
**n**	**17**	**31**	**83**	**187**	**46**	-	-
EQ-5D-5L	12.90 (12.00)	11.00 (9.70)	7.77 (7.54)	4.20 (7.65)	2.07 (4.02)	-	-
EQ-5D-5L + SE	19.10 (13.70)	17.90 (9.63)	12.80 (7.68)	7.11 (7.79)	2.54 (4.60)	2.36 (1.79–3.73)	2.36 (1.79–3.73)
EQ-5D-5L + SE + SR	20.40 (13.90)	19.20 (10.10)	13.40 (8.14)	7.22 (7.71)	2.64 (4.08)	1.15 (1.01–1.35)	2.72 (1.96–4.71)

*TI* tiredness bolt-on, *SE* self-confidence bolt-on, *SR* social relationships bolt-on, *RE* relative efficiency, *CI* Confidence interval, *LSS* mean level sum scores, *RSES* Rosenberg Self-Esteem Scale (RSES)

Scores less than 15 indicate low self-esteem on the RSES. * =2000 bootstrap samples with accelerated bias correction

## Discussion

This study investigated the psychometric properties of five existing bolt-ons for the EQ-5D-5L in women who had undergone or were planning MICPs. The EQ-5D-5L core dimensions showed a high ceiling in this population, with nearly half of the sample reporting ‘full health’ (11111 profile). However, almost half of the respondents in 11111 reported HRQoL problems on at least one bolt-on. Adding bolt-ons to the EQ-5D-5L improved its psychometric properties, including a reduction in ceiling and improved informativity, explanatory power for EQ VAS and known-groups validity. Furthermore, the self-confidence and social relationships bolt-ons showed good convergent validity with widely used and validated external measures, such as the RSES and BFNE-S. Overall, the tiredness and self-confidence bolt-ons performed particularly well considering multiple psychometric properties. However, the skin irritation bolt-on seemed less useful in this population.

A recent study assessed the psychometric performance of nine existing bolt-ons (including all five tested in the present study) for the EQ-5D-5L in a large general population sample in Hungary [33]. Compared to the general population, more respondents in the MICP population reported at least some problems with self-confidence (43% vs. 69%), social relationships (28% vs. 35%), tiredness (62% vs. 74%) and sleep (45% vs. 55%). However, similar proportion of respondents reported problems with skin irritation in the MICP population compared to the general population (18% vs. 19%). The difference is particularly notable for self-confidence, confirming the relevance of the self-confidence bolt-on for this MICP population. It is important to acknowledge that the comparison of these results is somewhat limited by the fact that the general population sample was collected during the COVID-19 pandemic, which may have affected psychosocial health.

Similarly to the abovementioned general population survey, the strongest correlation among any of the bolt-ons and EQ-5D-5L core dimensions was found between the self-confidence bolt-on and anxiety/depression [33]. This can be explained by the association of low self-confidence and mental illnesses such as anxiety and depression [54]. Nevertheless, qualitative studies (interviews and focus groups with psoriasis and atopic dermatitis patients) confirmed that self-confidence and anxiety/depression are conceptually different, and the self-confidence bolt-on extends the content covered by the core descriptive system [21, 23]. Our study demonstrated that the self-confidence bolt-on enhanced the psychometric performance of the EQ-5D-5L more effectively than the social relationships bolt-on.

The popularity of MICPs is increasing, and the demand for them is growing [55]. Moreover, the population in developed countries is aging rapidly [56]. In most countries, MICPs are not covered by health insurance, and therefore, patients finance these procedures out-of-pocket. These populations typically experience the most HRQoL problems in psychosocial aspects that the EQ-5D-5L might not fully capture. Our study is particularly important as it suggests that bolt-on dimensions can enhance the EQ-5D-5L’s sensitivity in this specific group. Although our study focused solely on psychometric properties and did not examine the impact of these additional dimensions on health utilities, previous research indicates that such bolt-ons may affect preferences [20, 35]. Thus, incorporating selected bolt-ons into the EQ-5D-5L has the potential to improve the outcomes of cost-utility analyses for MICPs, thereby better demonstrating the value of these interventions.

Our study has several limitations. First, convenience sampling was used, and only data from female respondents were analyzed. Therefore, the sample may not be fully representative of the population using MICPs in Hungary and may not be generalizable to males, older adults, or other demographic groups. Data collection primarily relied on a self-administered online survey, which collected self-reported clinical data that was not verified by a physician. The fixed order of standardized questionnaires, including the bolt-ons, may have introduced an order effect. Another limitation is the study’s cross-sectional design that did not allow to assess the responsiveness and test-retest reliability of EQ-5D-5L and bolt-ons. Future research is recommended to investigate these psychometric properties as well as the impact of bolt-ons on utility values.

## Conclusion

Relevant bolt-ons significantly enhance the psychometric performance of the EQ-5D-5L in women planning or undergoing MICPs, including reductions in ceiling and improvements in informativity and known-group validity. Based on our findings, the self-confidence and tiredness bolt-ons demonstrated the best performance and may, therefore, be recommended as primary bolt-on choices for this population.

## Electronic supplementary material

Below is the link to the electronic supplementary material.

Supplementary Material 1

## Data Availability

All data of this study are available from the corresponding author upon reasonable request.

## Code availability

N/A.

## References

[1] International Society of Aesthetic Plastic Surgery (ISAPS): International survey on aesthetic/cosme procedures. Available from: https://www.isaps.org/media/rxnfqibn/isaps-global-survey_2023.pdf (2023). Accessed 26 Jan 2025

[2] Ogden, S., Griffiths, T.W.: A review of minimally invasive cosmetic procedures. Br. J. Dermatol. **159**, 1036–1050 (2008). 10.1111/j.1365-2133.2008.08845.x 18823403 10.1111/j.1365-2133.2008.08845.x

[3] Chuang, J., Barnes, C., Wong, B.J.F.: Overview of facial plastic surgery and current developments. Surg. J. **2**, e17–28 (2016). 10.1055/s-0036-1572360 10.1055/s-0036-1572360 PMC5553462 28824978

[4] Maisel, A., Waldman, A., Furlan, K., Weil, A., Sacotte, K., Lazaroff, J.M., et al.: Self-reported patient motivations for seeking cosmetic procedures. JAMA Dermatol. **154**, 1167–1174 (2018). 10.1001/jamadermatol.2018.2357 30140900 10.1001/jamadermatol.2018.2357 PMC6233736

[5] Waldman, A., Maisel, A., Weil, A., Iyengar, S., Sacotte, K., Lazaroff, J.M., et al.: Patients believe that cosmetic procedures affect their quality of life: An interview study of patient-reported motivations. J. Am. Acad. Dermatol. **80**, 1671–1681 (2019). 10.1016/j.jaad.2019.01.059 30710607 10.1016/j.jaad.2019.01.059

[6] Rudolph, C., Hladik, C., Stroup, D.F., Frank, K., Gotkin, R.H., Dayan, S.H., et al.: Are cosmetic procedures comparable to antidepressive medication for Quality-of-Life improvements?? A systematic review and controlled Meta-Analysis. Facial Plast. Surg. **35**, 549–558 (2019). 10.1055/s-0039-1697030 31563125 10.1055/s-0039-1697030

[7] Imadojemu, S., Sarwer, D.B., Percec, I., Sonnad, S.S., Goldsack, J.E., Berman, M., et al.: Influence of surgical and minimally invasive facial cosmetic procedures on psychosocial outcomes: A systematic review. JAMA Dermatol. **149**, 1325–1333 (2013). 10.1001/jamadermatol.2013.6812 24068036 10.1001/jamadermatol.2013.6812

[8] Sobanko, J.F., Dai, J., Gelfand, J.M., Sarwer, D.B., Percec, I.: Prospective cohort study investigating changes in body image, quality of life, and Self-Esteem following minimally invasive cosmetic procedures. Dermatol. Surg. **44**, 1121 (2018). 10.1097/DSS.0000000000001523 29659404 10.1097/DSS.0000000000001523

[9] McKeown, D.J.: Impact of minimally invasive aesthetic procedures on the psychological and social dimensions of health. Plast. Reconstr. Surg. – Glob Open. **9**, e3578 (2021). 10.1097/GOX.0000000000003578 33936919 10.1097/GOX.0000000000003578 PMC8081460

[10] Pereira, I.N., Hassan, H.: Impact of botulinum toxin for facial aesthetics on psychological well-being and quality of life: Evidence-based review. J. Plast. Reconstr. Aesthet. Surg. **75**, 4450–4463 (2022). 10.1016/j.bjps.2022.08.063 36274011 10.1016/j.bjps.2022.08.063

[11] Ware, J.E., Sherbourne, C.D.: The MOS 36-item short-form health survey (SF-36). I. Conceptual framework and item selection. Med. Care. **30**, 473–483 (1992) 1593914

[12] Sandman, L., Hansson, E.: An ethics analysis of the rationale for publicly funded plastic surgery. BMC Med. Ethics. **21**, 94 (2020). 10.1186/s12910-020-00539-6 33008385 10.1186/s12910-020-00539-6 PMC7531084

[13] Kennedy-Martin, M., Slaap, B., Herdman, M., van Reenen, M., Kennedy-Martin, T., Greiner, W., et al.: Which multi-attribute utility instruments are recommended for use in cost-utility analysis? A review of National health technology assessment (HTA) guidelines. Eur. J. Health Econ. **21**, 1245–1257 (2020). 10.1007/s10198-020-01195-8 32514643 10.1007/s10198-020-01195-8 PMC7561556

[14] Rencz, F., Gulácsi, L., Drummond, M., Golicki, D., Prevolnik Rupel, V., Simon, J., et al.: EQ-5D in central and Eastern Europe: 2000–2015. Qual. Life Res. **25**, 2693–2710 (2016). 10.1007/s11136-016-1375-6 27472992 10.1007/s11136-016-1375-6

[15] Feng, Y.-S., Kohlmann, T., Janssen, M.F., Buchholz, I.: Psychometric properties of the EQ-5D-5L: A systematic review of the literature. Qual. Life Res. **30**, 647–673 (2021). 10.1007/s11136-020-02688-y 33284428 10.1007/s11136-020-02688-y PMC7952346

[16] Herdman, M., Gudex, C., Lloyd, A., Janssen, M., Kind, P., Parkin, D., et al.: Development and preliminary testing of the new five-level version of EQ-5D (EQ-5D-5L). Qual. Life Res. **20**, 1727–1736 (2011). 10.1007/s11136-011-9903-x 21479777 10.1007/s11136-011-9903-x PMC3220807

[17] Shah, K.K., Mulhern, B., Longworth, L., Janssen, M.F.: Views of the UK general public on important aspects of health not captured by EQ-5D. Patient. **10**, 701–709 (2017). 10.1007/s40271-017-0240-1 28409481 10.1007/s40271-017-0240-1

[18] Mulhern, B.J., Sampson, C., Haywood, P., Addo, R., Page, K., Mott, D., et al.: Criteria for developing, assessing and selecting candidate EQ-5D bolt-ons. Qual. Life Res. **31**, 3041–3048 (2022). 10.1007/s11136-022-03138-7 35486216 10.1007/s11136-022-03138-7 PMC9470642

[19] Geraerds, A.J.L.M., Bonsel, G.J., Janssen, M.F., Finch, A.P., Polinder, S., Haagsma, J.A.: Methods used to identify, test, and assess impact on preferences of Bolt-Ons: A systematic review. Value Health. **24**, 901–916 (2021). 10.1016/j.jval.2020.12.011 34119088 10.1016/j.jval.2020.12.011

[20] Swinburn, P., Lloyd, A., Boye, K.S., Edson-Heredia, E., Bowman, L., Janssen, B.: Development of a Disease-Specific version of the EQ-5D-5L for use in patients suffering from psoriasis: Lessons learned from a feasibility study in the UK. Value Health. **16**, 1156–1162 (2013). 10.1016/j.jval.2013.10.003 24326169 10.1016/j.jval.2013.10.003

[21] Rencz, F., Mukuria, C., Bató, A., Poór, A.K., Finch, A.P.: A qualitative investigation of the relevance of skin irritation and self-confidence bolt-ons and their conceptual overlap with the EQ-5D in patients with psoriasis. Qual. Life Res. **31**, 3049–3060 (2022). 10.1007/s11136-022-03141-y 35471487 10.1007/s11136-022-03141-y PMC9039271

[22] Pickard, A.S., Gooderham, M., Hartz, S., Nicolay, C.: EQ-5D health utilities: Exploring ways to improve upon responsiveness in psoriasis. J. Med. Econ. **20**, 19–27 (2017). 10.1080/13696998.2016.1219359 27471948 10.1080/13696998.2016.1219359

[23] Szlávicz, E., Szabó, Á., Kinyó, Á., Szeiffert, A., Bancsók, T., Brodszky, V., et al.: Content validity of the EQ-5D-5L with skin irritation and self-confidence bolt-ons in patients with atopic dermatitis: A qualitative think-aloud study. Qual. Life Res. **33**, 101–111 (2024). 10.1007/s11136-023-03519-6 37787930 10.1007/s11136-023-03519-6 PMC10784357

[24] Sussex, A.-K., Rencz, F., Gaydon, M., Lloyd, A., Gallop, K.: Exploring the content validity of the EQ-5D-5L and four bolt-ons (skin irritation, self-confidence, sleep, social relationships) in atopic dermatitis and chronic urticaria. Qual. Life Res. (2024). 10.1007/s11136-024-03875-x 39673587 10.1007/s11136-024-03875-x PMC11982113

[25] Plázár, D., Metyovinyi, Z., Medvecz, M., Rencz, F.: Qualitative evidence on EQ-5D-5L skin irritation and self-confidence bolt-ons in Darier’s disease and Hailey-Hailey disease. Qual. Life Res. (2024). 10.1007/s11136-024-03871-1 39704914 10.1007/s11136-024-03871-1 PMC11982107

[26] Hamilton, N.A., Nelson, C.A., Stevens, N., Kitzman, H.: Sleep and psychological well-being. Soc. Indic. Res. **82**, 147–163 (2007). 10.1007/s11205-006-9030-1

[27] Chen, G., Olsen, J.A.: Filling The psycho-social gap in The EQ-5D: The empirical support for four bolt-on dimensions. Qual. Life Res. **29**, 3119–3129 (2020). 10.1007/s11136-020-02576-5 32648198 10.1007/s11136-020-02576-5 PMC7591404

[28] Chen, G., Olsen, J.A.: Extending The EQ-5D: The case for a complementary set of 4 psycho-social dimensions. Qual. Life Res. **32**, 495–505 (2023). 10.1007/s11136-022-03243-7 36125601 10.1007/s11136-022-03243-7 PMC9486772

[29] Crowner, B.E., Torres-Russotto, D., Carter, A.R., Racette, B.A.: Systemic weakness after therapeutic injections of botulinum toxin A: A case series and review of the literature. Clin. Neuropharmacol. **33**, 243–247 (2010). 10.1097/WNF.0b013e3181f5329e 20852412 10.1097/WNF.0b013e3181f5329e PMC3563356

[30] Pearl, R.L., Percec, I.: Ageism and health in patients undergoing cosmetic procedures. Aesthet. Surg. J. **39**, NP288–NP292 (2019). 10.1093/asj/sjy283 30346472 10.1093/asj/sjy283 PMC6587926

[31] Haagsma, J., et al.: POPCORN: Longitudinal effects of the COVID-19 pandemic on individual’s health-related quality of life. EQ Project 238–RA. (2021)

[32] Dewilde, S., Phillips, G., Paci, S., De Ruyck, F., Tollenaar, N.H., Janssen, M.F.: The burden patients with myasthenia Gravis experience in terms of breathing, fatigue, sleep, mental health, discomfort and usual activities in comparison to the general population. Adv. Ther. **41**, 271–291 (2024). 10.1007/s12325-023-02704-w 37921955 10.1007/s12325-023-02704-w PMC10796601

[33] Rencz, F., Janssen, M.F.: Testing the psychometric properties of 9 Bolt-Ons for the EQ-5D-5L in a general population sample. Value Health. **27**, 943–954 (2024). 10.1016/j.jval.2024.03.2195 10.1016/j.jval.2024.03.2195 38599517

[34] Longworth, L., Yang, Y., Young, T., Mulhern, B., Hernández Alava, M., Mukuria, C., et al.: Use of generic and condition-specific measures of health-related quality of life in NICE decision-making: A systematic review, statistical modelling and survey 2014. Health Technol. Assess. **18**, 1–224 (2014). 10.3310/hta18090 10.3310/hta18090 PMC4780954 24524660

[35] Yang, Y., Brazier, J., Tsuchiya, A.: Effect of adding a sleep dimension to the EQ-5D descriptive system: A Bolt-On experiment. Med. Decis. Mak. **34**, 42–53 (2014). 10.1177/0272989X13480428 10.1177/0272989X13480428 23525702

[36] Perneger, T.V., Courvoisier, D.S.: Exploration of health dimensions to be included in multi-attribute health-utility assessment. Int. J. Qual. Health Care. **23**, 52–59 (2011). 10.1093/intqhc/mzq068 21084324 10.1093/intqhc/mzq068

[37] Finch, A.P., Brazier, J., Mukuria, C.: Selecting Bolt-on dimensions for the EQ-5D: Testing the impact of hearing, sleep, cognition, energy, and relationships on preferences using pairwise choices. Med. Decis. Mak. **41**, 89–99 (2021). 10.1177/0272989X20969686 10.1177/0272989X20969686 PMC7780267 33256502

[38] Angyal, M.M., Janssen, M.F., Lakatos, P.L., Brodszky, V., Rencz, F.: The added value of the cognition, dining, Gastrointestinal problems, sleep and tiredness bolt-on dimensions to the EQ-5D-5L in patients with coeliac disease. Eur. J. Health Econ. (2024). 10.1007/s10198-024-01719-6 39212881 10.1007/s10198-024-01719-6 PMC11937053

[39] Xu, R.H., Rencz, F., Sun, R., Dong, D., Zhang, S.: Development and testing the psychometric properties of 20 bolt-on items for the EQ-5D-5L across 31 rare diseases. Value Health. (2025). 10.1016/j.jval.2025.01.006 10.1016/j.jval.2025.01.006 39880195

[40] Dewilde, S., Janssen, M.F., Mulhern, B., Rencz, F.: MSR129 psychometric properties of six EQ-5D-5L Bolt-Ons in a rare disease population: A multinational, longitudinal study. Value Health. **27**, S463 (2024). 10.1016/j.jval.2024.10.2363

[41] Rencz, F., Brodszky, V., Gulácsi, L., Golicki, D., Ruzsa, G., Pickard, A.S., et al.: Parallel valuation of the EQ-5D-3L and EQ-5D-5L by time Trade-Off in Hungary. Value Health. **23**, 1235–1245 (2020). 10.1016/j.jval.2020.03.019 32940242 10.1016/j.jval.2020.03.019

[42] Sallay, V., Martos, T., Földvári, M., Szabó, T., Ittzés, A.: A Rosenberg önértékelés Skála (RSES-H): Alternatív Fordítás, strukturális invariancia És validitás [Hungarian version of the Rosenberg Self-esteem Scale (RSES-H): An alternative translation, structural invariance, and validity]. Mentálhig Es Pszichoszomatika. **15**, 259–275 (2014). 10.1556/Mental.15.2014.3.7

[43] Rosenberg, M.: Rosenberg Self-Esteem scale (RSES) [Database record]. APA PsycTests. (1965). 10.1037/t01038-000

[44] Donnellan, M.B., Trzesniewski, K.H., Robins, R.W.: Chapter 6 - Measures of Self-Esteem. In: Boyle, G.J., Saklofske, D.H., Matthews, G. (eds.) Meas. Personal. Soc. Psychol. Constr, pp. 131–157. Academic, San Diego (2015). 10.1016/B978-0-12-386915-9.00006-1

[45] Isomaa, R., Väänänen, J.-M., Fröjd, S., Kaltiala, R., Marttunen, M.: How low is low? low Self-Esteem as an Indicator of internalizing psychopathology in adolescence. Health Educ. Behav. **40**, 392–399 (2013). 10.1177/1090198112445481 22872582 10.1177/1090198112445481

[46] Perczel-Forintos, D., Kresznerits, S.: [Social anxiety and self-esteem: Hungarian validation of the brief fear of negative evaluation Scale - Straightforward items]. Orv Hetil. **158**, 843–850 (2017). 10.1556/650.2017.30755 28561634 10.1556/650.2017.30755

[47] Rodebaugh, T.L., Woods, C.M., Thissen, D.M., Heimberg, R.G., Chambless, D.L., Rapee, R.M.: More information from fewer questions: The factor structure and item properties of The original and brief fear of negative evaluation scale. Psychol. Assess. **16**, 169–181 (2004). 10.1037/1040-3590.16.2.169 15222813 10.1037/1040-3590.16.2.169

[48] Development of the World Health Organization WHOQOL-BREF quality of life assessment: The WHOQOL group. Psychol. Med. **28**, 551–558 (1998). 10.1017/s0033291798006667 9626712 10.1017/s0033291798006667

[49] Shannon, C.E.: A mathematical theory of communication. Bell Syst. Tech. J. **27**, 379–423 (1948). 10.1002/j.1538-7305.1948.tb01338.x

[50] Bas Janssen, M.F., Birnie, E., Bonsel, G.J.: Evaluating the discriminatory power of EQ-5D, HUI2 and HUI3 in a US general population survey using Shannon’s indices. Qual. Life Res. **16**, 895–904 (2007). 10.1007/s11136-006-9160-6 17294285 10.1007/s11136-006-9160-6 PMC1915610

[51] Rosenberg, M.: The association between self-esteem and anxiety. J. Psychiatr Res. **1**, 135–152 (1962). 10.1016/0022-3956(62)90004-3 13974903 10.1016/0022-3956(62)90004-3

[52] Evans, J.D.: Straightforward Statistics for the Behavioral Sciences. Thomson Brooks/Cole Publishing Co, Belmont, CA, US (1996)

[53] Rencz, F., Brodszky, V., Janssen, M.F.: A direct comparison of the measurement properties of EQ-5D-5L, PROMIS-29 + 2 and PROMIS global health instruments and EQ-5D-5L and PROPr utilities in a general population sample. Value Health. **26**, 1045–1056 (2023). 10.1016/j.jval.2023.02.002 36804583 10.1016/j.jval.2023.02.002

[54] Sowislo, J.F., Orth, U.: Does low self-esteem predict depression and anxiety? A meta-analysis of longitudinal studies. Psychol. Bull. **139**, 213–240 (2013). 10.1037/a0028931 22730921 10.1037/a0028931

[55] Wang, J.V., Akintilo, L., Geronemus, R.G.: Growth of cosmetic procedures in millennials: A 4.5-year clinical review. J. Cosmet. Dermatol. **19**, 3210–3212 (2020). 10.1111/jocd.13768 33030801 10.1111/jocd.13768

[56] Cheng, X., Yang, Y., Schwebel, D.C., Liu, Z., Li, L., Cheng, P., et al.: Population ageing and mortality during 1990–2017: A global decomposition analysis. PLOS Med. **17**, e1003138 (2020). 10.1371/journal.pmed.1003138 32511229 10.1371/journal.pmed.1003138 PMC7279585

