# Supplementary material for: Psychometric benefits of adding bolt-ons to the EQ-5D-5L in populations undergoing minimally invasive cosmetic procedures
Source: Eur J Health Econ. 2025 Mar 13;26(7):1233–47. doi: 10.1007/s10198-025-01772-9 (PMC12432065; doi:10.1007/s10198-025-01772-9)
Supplement: Supplementary file 1 — Supplementary Material 1 [file 10198_2025_1772_MOESM1_ESM.docx]

**Online Resource 1 The five bolt-on items tested in this study**

| RELATIONSHIPS |
| --- |
| I have no problems with social relationships |
| I have slight problems with social relationships |
| I have moderate problems with social relationships |
| I have severe problems with social relationships |
| I have extreme problems with social relationships |
| SLEEP |
| I have no problems sleeping |
| I have slight problems sleeping |
| I have moderate problems sleeping |
| I have severe problems sleeping |
| I have extreme problems sleeping |
| TIREDNESS |
| I am not tired |
| I am slightly tired |
| I am moderately tired |
| I am severely tired |
| I am extremely tired |
| SKIN IRRITATION *(e.g. itching)* |
| I have no itching |
| I have slight itching |
| I have moderate itching |
| I have severe itching |
| I have extreme itching |
| SELF-CONFIDENCE |
| I have no problems with self-confidence |
| I have slight problems with self-confidence |
| I have moderate problems with self -confidence |
| I have severe problems with self-confidence |
| I have extreme problems with self-confidence |

***© EuroQol Research Foundation. EQ-5D™ is a trademark of the EuroQol Research Foundation.***

***This is a modified EQ-5D. Reproduced by permission of EuroQol Research Foundation. Reproduction of this version is not allowed. For reproduction, use, or modification of the EQ-5D (any version), please register your study by using the online EQ***

***registration page:*** [***www.euroqol.org***](http://www.euroqol.org)

**Online Resource 2 study flow-chart**

Participants opened the questionnaire **(n=942)**

Participants completed the questionnaire **(n=387)**

Participants included in the data analysis **(n=364)**

Excluded **(n=555)**

- Refused to accept the informed consent **(n=161)**
- Withdrew in the middle of the questionnaire **(n=394)**

Excluded **(n=23)**

- Filled out the questionnaire in under 6 minutes **(n=5)**
- Male and non-binary participants **(n=18)**

**Online Resource 3 Responses on the Rosenberg self-esteem scale (RSES)**

| Items | **Strongly Agree** | | **Agree** | | **Disagree** | | **Strongly Disagree** | |
| --- | --- | --- | --- | --- | --- | --- | --- | --- |
|  | **n** | **%** | **n** | **%** | **n** | **%** | **n** | **%** |
| **Total sample (n=364)** |  |  |  |  |  |  |  |  |
| Item 1 - On the whole, I am satisfied with myself. | 89 | 24.5 | 169 | 46.4 | 88 | 24.2 | 18 | 4.9 |
| Item 2 - At times I think I am no good at all. * | 29 | 8.0 | 86 | 23.6 | 101 | 27.7 | 148 | 40.7 |
| Item 3 - I feel that I have a number of good qualities. | 188 | 51.6 | 152 | 41.8 | 20 | 5.5 | 4 | 1.1 |
| Item 4 - I am able to do things as well as most other people. | 184 | 50.5 | 150 | 41.2 | 28 | 7.7 | 2 | 0.5 |
| Item 5 - I feel I do not have much to be proud of. * | 25 | 6.9 | 51 | 14.0 | 109 | 29.9 | 179 | 49.2 |
| Item 6 - I certainly feel useless at times. * | 34 | 9.3 | 105 | 28.8 | 110 | 30.2 | 115 | 31.6 |
| Item 7 - I feel that I'm a person of worth, at least on an equal plane with others. | 198 | 54.4 | 136 | 37.4 | 25 | 6.9 | 5 | 1.4 |
| Item 8 - I wish I could have more respect for myself. * | 62 | 17.0 | 111 | 30.5 | 75 | 20.6 | 116 | 31.9 |
| Item 9 - All in all, I am inclined to feel that I am a failure. * | 23 | 6.3 | 74 | 20.3 | 110 | 30.2 | 157 | 43.1 |
| Item 10 - I take a positive attitude toward myself. | 96 | 26.4 | 181 | 49.7 | 78 | 21.4 | 9 | 2.5 |
| **Group 1 (undergone, n=215)** |  |  |  |  |  |  |  |  |
| Item 1 - On the whole, I am satisfied with myself. | 57 | 26.5 | 108 | 50.2 | 40 | 18.6 | 10 | 4.7 |
| Item 2 - At times I think I am no good at all. * | 9 | 4.2 | 42 | 19.5 | 70 | 32.6 | 94 | 43.7 |
| Item 3 - I feel that I have a number of good qualities. | 121 | 56.3 | 85 | 39.5 | 5 | 2.3 | 4 | 1.9 |
| Item 4 - I am able to do things as well as most other people. | 113 | 52.6 | 86 | 40.0 | 14 | 6.5 | 2 | 0.9 |
| Item 5 - I feel I do not have much to be proud of. * | 19 | 8.8 | 25 | 11.6 | 55 | 25.6 | 116 | 54.0 |
| Item 6 - I certainly feel useless at times. * | 12 | 5.6 | 56 | 26.0 | 72 | 33.5 | 75 | 34.9 |
| Item 7 - I feel that I'm a person of worth, at least on an equal plane with others. | 128 | 59.5 | 73 | 34.0 | 10 | 4.7 | 4 | 1.9 |
| Item 8 - I wish I could have more respect for myself. * | 34 | 15.8 | 58 | 27.0 | 49 | 22.8 | 74 | 34.4 |
| Item 9 - All in all, I am inclined to feel that I am a failure. * | 8 | 3.7 | 36 | 16.7 | 66 | 30.7 | 105 | 48.8 |
| Item 10 - I take a positive attitude toward myself. | 59 | 27.4 | 118 | 54.9 | 34 | 15.8 | 4 | 1.9 |
| **Group 2 (planning, n=149)** |  |  |  |  |  |  |  |  |
| Item 1 - On the whole, I am satisfied with myself. | 32 | 21.5 | 61 | 40.9 | 48 | 32.2 | 8 | 5.4 |
| Item 2 - At times I think I am no good at all. * | 20 | 13.4 | 44 | 29.5 | 31 | 20.8 | 54 | 36.2 |
| Item 3 - I feel that I have a number of good qualities. | 67 | 45.0 | 67 | 45.0 | 15 | 10.1 | 0 | 0.0 |
| Item 4 - I am able to do things as well as most other people. | 71 | 47.7 | 64 | 43.0 | 14 | 9.4 | 0 | 0.0 |
| Item 5 - I feel I do not have much to be proud of. * | 6 | 4.0 | 26 | 17.4 | 54 | 36.2 | 63 | 42.3 |
| Item 6 - I certainly feel useless at times. * | 22 | 14.8 | 49 | 32.9 | 38 | 25.5 | 40 | 26.8 |
| Item 7 - I feel that I'm a person of worth, at least on an equal plane with others. | 70 | 47.0 | 63 | 42.3 | 15 | 10.1 | 1 | 0.7 |
| Item 8 - I wish I could have more respect for myself. * | 28 | 18.8 | 53 | 35.6 | 26 | 17.4 | 42 | 28.2 |
| Item 9 - All in all, I am inclined to feel that I am a failure. * | 15 | 10.1 | 38 | 25.5 | 44 | 29.5 | 52 | 34.9 |
| Item 10 - I take a positive attitude toward myself. | 37 | 24.8 | 63 | 42.3 | 44 | 29.5 | 5 | 3.4 |

RSES Rosenberg Self-Esteem Scale

Values may range from 0 to 30 on the Rosenberg Self-Esteem Scale. Negatively worded items are marked with asterisks.

**Online Resource 4 Responses on the Brief version of the Fear of Negative Evaluation Scale - Straightforward Items (BFNE-S)**

| Items | **Not at all characterstic of me** | | **Slightly characteristic of me** | | **Moderately characteristic of me** | | **Very characteristic of me** | | **Extremely characteristic of me** | |
| --- | --- | --- | --- | --- | --- | --- | --- | --- | --- | --- |
|  | **n** | **%** | **n** | **%** | **n** | **%** | **n** | **%** | **n** | **%** |
| **Total sample (n=364)** |  |  |  |  |  |  |  |  |  |  |
| Item 1 - I worry about what other people will think of me even when I know it doesn't make any difference. | 89 | 24.5 | 155 | 42.6 | 38 | 10.4 | 49 | 13.5 | 33 | 9.1 |
| Item 2 - I am frequently afraid of other people noticing my shortcomings. | 92 | 25.3 | 150 | 41.2 | 37 | 10.2 | 48 | 13.2 | 37 | 10.2 |
| Item 3 - I am afraid others will not approve of me. | 145 | 39.8 | 145 | 39.8 | 33 | 9.1 | 25 | 6.9 | 16 | 4.4 |
| Item 4 - I am afraid that other people will find fault with me. | 124 | 34.1 | 145 | 39.8 | 32 | 8.8 | 34 | 9.3 | 29 | 8.0 |
| Item 5 - When I am talking to someone, I worry about what they may be thinking about me. | 134 | 36.8 | 135 | 37.1 | 37 | 10.2 | 36 | 9.9 | 22 | 6.0 |
| Item 6 - I am usually worried about what kind of impression I make. | 109 | 29.9 | 136 | 37.4 | 56 | 15.4 | 28 | 7.7 | 35 | 9.6 |
| Item 7 - Sometimes I think I am too concerned with what other people think of me. | 132 | 36.3 | 106 | 29.1 | 47 | 12.9 | 35 | 9.6 | 44 | 12.1 |
| Item 8 - I often worry that I will say or do the wrong things. | 53 | 14.6 | 148 | 40.7 | 60 | 16.5 | 56 | 15.4 | 47 | 12.9 |
| **Group 1 (undergone, n=215)** |  |  |  |  |  |  |  |  |  |  |
| Item 1 - I worry about what other people will think of me even when I know it doesn't make any difference. | 60 | 27.9 | 95 | 44.2 | 22 | 10.2 | 25 | 11.6 | 13 | 6.0 |
| Item 2 - I am frequently afraid of other people noticing my shortcomings. | 62 | 28.8 | 95 | 44.2 | 17 | 7.9 | 26 | 12.1 | 15 | 7.0 |
| Item 3 - I am afraid others will not approve of me. | 94 | 43.7 | 87 | 40.5 | 17 | 7.9 | 11 | 5.1 | 6 | 2.8 |
| Item 4 - I am afraid that other people will find fault with me. | 80 | 37.2 | 86 | 40.0 | 21 | 9.8 | 17 | 7.9 | 11 | 5.1 |
| Item 5 - When I am talking to someone, I worry about what they may be thinking about me. | 91 | 42.3 | 82 | 38.1 | 19 | 8.8 | 15 | 7.0 | 8 | 3.7 |
| Item 6 - I am usually worried about what kind of impression I make. | 71 | 33.0 | 89 | 41.4 | 33 | 15.3 | 10 | 4.7 | 12 | 5.6 |
| Item 7 - Sometimes I think I am too concerned with what other people think of me. | 88 | 40.9 | 61 | 28.4 | 26 | 12.1 | 19 | 8.8 | 21 | 9.8 |
| Item 8 - I often worry that I will say or do the wrong things. | 35 | 16.3 | 102 | 47.4 | 32 | 14.9 | 27 | 12.6 | 19 | 8.8 |
| **Group 2 (planning, n=149)** |  |  |  |  |  |  |  |  |  |  |
| Item 1 - I worry about what other people will think of me even when I know it doesn't make any difference. | 29 | 19.5 | 60 | 40.3 | 16 | 10.7 | 24 | 16.1 | 20 | 13.4 |
| Item 2 - I am frequently afraid of other people noticing my shortcomings. | 30 | 20.1 | 55 | 36.9 | 20 | 13.4 | 22 | 14.8 | 22 | 14.8 |
| Item 3 - I am afraid others will not approve of me. | 51 | 34.2 | 58 | 38.9 | 16 | 10.7 | 14 | 9.4 | 10 | 6.7 |
| Item 4 - I am afraid that other people will find fault with me. | 44 | 29.5 | 59 | 39.6 | 11 | 7.4 | 17 | 11.4 | 18 | 12.1 |
| Item 5 - When I am talking to someone, I worry about what they may be thinking about me. | 43 | 28.9 | 53 | 35.6 | 18 | 12.1 | 21 | 14.1 | 14 | 9.4 |
| Item 6 - I am usually worried about what kind of impression I make. | 38 | 25.5 | 47 | 31.5 | 23 | 15.4 | 18 | 12.1 | 23 | 15.4 |
| Item 7 - Sometimes I think I am too concerned with what other people think of me. | 44 | 29.5 | 45 | 30.2 | 21 | 14.1 | 16 | 10.7 | 23 | 15.4 |
| Item 8 - I often worry that I will say or do the wrong things. | 18 | 12.1 | 46 | 30.9 | 28 | 18.8 | 29 | 19.5 | 28 | 18.8 |

BFNE-S Brief version of the Fear of Negative Evaluation Scale - Straightforward Items

Values may range from 8 to 40 on the Brief Fear of Negative Evaluation Scale - Straightforward Items
